# Vaccine Uptake in the US After Full Food and Drug Administration Approval of the BNT162b2 mRNA COVID-19 Vaccine

**DOI:** 10.1001/jamanetworkopen.2022.6108

**Published:** 2022-04-06

**Authors:** Elise Vandersteen Bailey, Fernando A. Wilson

**Affiliations:** 1 Department of Population Health Sciences, University of Utah, Salt Lake City; 2 College of Social and Behavioral Science, University of Utah, Salt Lake City; 3 Matheson Center for Health Care Studies, University of Utah, Salt Lake City; 4 Department of Economics, University of Utah, Salt Lake City

## Abstract

This cross-sectional study examines the association of full approval of the BNT162b2 mRNA vaccine by the US Food and Drug Administration (FDA) with subsequent US uptake of COVID-19 vaccines of any kind.

## Introduction

On August 23, 2021, the US Food and Drug Administration (FDA) gave its first full approval of a COVID-19 vaccine, the BNT162b2 mRNA vaccine (Pfizer-BioNTech).^1^ Previously, a Kaiser Family Foundation survey^2^ found that many unvaccinated individuals were concerned the vaccine was unsafe, and some remained unvaccinated because they did not trust the government. It is unclear whether FDA approval allayed this hesitancy. To examine this notion, we conducted a cross-sectional analysis of the association between the FDA full approval of BNT162b2 mRNA vaccine with subsequent US COVID-19 vaccinations of any kind.

## Methods

Daily US vaccination data were obtained from the Centers for Disease Control and Prevention’s COVID Data Tracker.^3^ Daily UK vaccinations came from the Coronavirus (COVID-19) in the UK website.^4^ The vaccination trend in the United Kingdom (UK) was used as a covarying series, which was combined with information about the preapproval trend in the US to create a synthetic control group against which observed US vaccination counts were compared. The period for analysis began 30 days before full FDA approval (July 25, 2021) and ended the day before the Biden administration’s vaccine mandate announcement on September 9, 2021. Because we used publicly available, deidentified data, this study is not viewed as human participants research by the Department of Health and Human Services; thus, no institutional review board approval or informed consent was sought, in accordance with 45 CFR §46. This study follows the Strengthening the Reporting of Observational Studies in Epidemiology (STROBE) reporting guideline.

In this analysis, which used bayesian structural time-series modeling, a counterfactual time trend that would have been observed without the impact of an intervention was constructed using a synthetic control. More information on the analytical approach are available in the eAppendix in the Supplement and elsewhere.^5^ We separately analyzed daily numbers of dose-agnostic vaccinations, first doses, and series-completing doses using R Studio statistical software version 2020 (RStudio Team). We generated 95% credible intervals (CrIs) using Markov Chain Monte Carlo methods. *P* < .05 in 2-sided hypothesis tests was considered significant.

## Results

In this analysis of daily dose-agnostic vaccinations, FDA approval was associated with an increase of 3.51 million cumulative vaccinations (95% CrI, 1.73 million to 5.26 million) over the study period, a 36% relative increase (Table and Figure). However, FDA approval was also associated with 1.2 million fewer cumulative first-dose vaccinations (95% CrI, −2.10 million to −0.36 million) compared with the counterfactual, a 16% relative decrease. In contrast, series-completing vaccinations increased after approval by 77%. Sensitivity analyses were conducted around inclusion of Labor Day; results were not sensitive to the choice (approval was associated, again, with a 36% cumulative increase in dose-agnostic vaccinations). Results were also substantively similar when excluding a covarying series from the analysis (approval was associated with a 20% cumulative increase in dose-agnostic vaccinations).

**Table.** Estimated Cumulative Association Between the Food and Drug Administration Approval of the BNT162b2 COVID-19 Vaccine and the Number of Daily COVID-19 Vaccinations Administered in the US, July 25, 2021, to September 9, 2021^a^

Measure	Total (95% CrI)
Dose-agnostic vaccinations	
Cumulative total after approval	
Observed, No.	13 239 759
Estimated counterfactual	9 778 314 (8 029 117 to 11 563 035)
Difference between observed and counterfactual	
Absolute	3 507 052 (1 731 416 to 5 256 249)
Relative, % (95% CI)	36 (17 to 53)
First doses	
Cumulative total after approval	
Observed, No.	6 216 950
Estimated counterfactual	7 411 478 (6 575 823 to 8 314 049)
Difference between observed and counterfactual	
Absolute^b^	−1 194 528 (−2 097 099 to −358 873)
Relative, % (95% CI)	−16 (−29 to −4.8)
Series-completing doses	
Cumulative total after approval	
Observed, No.	6 106 064
Estimated counterfactual	3 447 844 (2 508 240 to 4 348 668)
Difference between observed and counterfactual	
Absolute^b^	2 658 220 (1 757 396 to 3 597 834)
Relative, % (95% CI)	77 (51 to 104)

Abbreviation: CrI, credible interval.

^a^ This includes all available COVID-19 vaccinations at the time.

^b^ The absolute differences between observed and counterfactual in the first doses and the series-completing doses do not sum to the absolute difference between observed and counterfactual in dose-agnostic vaccinations because the 2 dose groups are not mutually exclusive. JNJ-78436735 (Johnson and Johnson/Janssen) vaccinations are single-dose and, therefore, belong to both groups.

**Figure.**
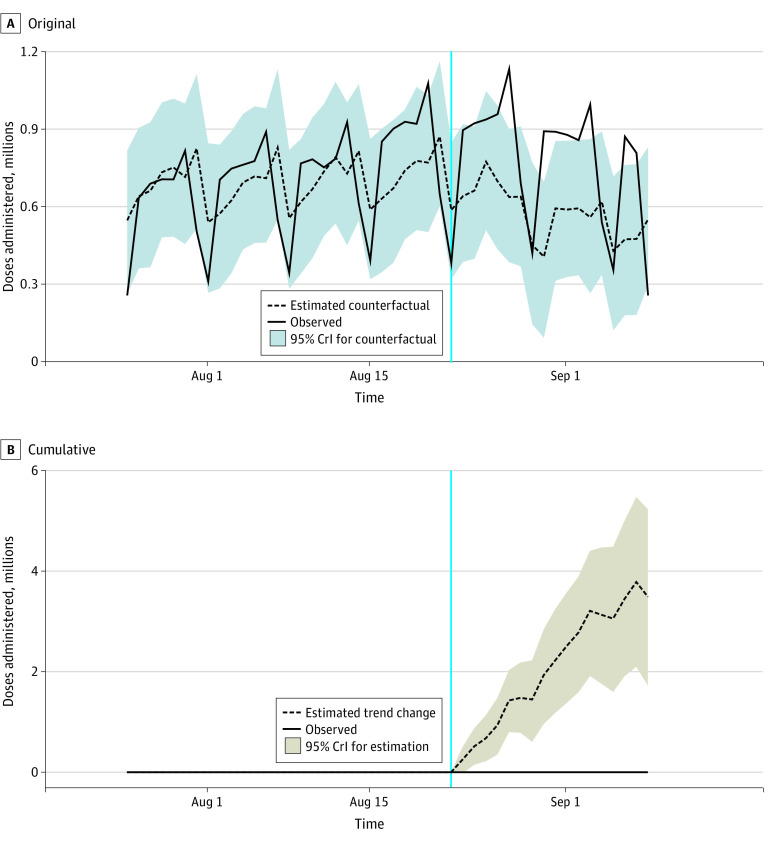
Analysis of the Association Between Food and Drug Administration (FDA) Approval of the BNT162b2 mRNA COVID-19 Vaccine With Subsequent Dose-Agnostic US COVID-19 Vaccinations Panel A shows the observed data (solid line) along with the counterfactual trend (dashed line). Panel B shows the cumulative difference between the observed data (solid line) and the counterfactual (dashed line) after FDA approval on August 23, 2021, which is denoted by the vertical cyan line. Shaded areas denote 95% credible intervals (CrIs).

## Discussion

To our knowledge, this cross-sectional study is the first to examine the association of the FDA’s full approval of the BNT162b2 mRNA COVID-19 vaccine with subsequent US COVID-19 vaccinations. We estimated that approval was significantly associated with an increase in the overall number of vaccines administered; however, this appears to be largely related to an increase in series completions after approval. Our analysis suggests that approval may have been associated with a decrease in the number of first doses administered compared with what would have been expected without approval. Potential limitations of this study include the possibility of spillover effects and the relative shortness of the postintervention period considered.

Additional research is needed to understand the underlying reasons for this differential response to FDA approval. Individuals who have not received any dose of a COVID-19 vaccine may be behaviorally distinct from those who have chosen to receive at least 1 dose. The results in the unvaccinated population may be related to negative perceptions of government sources of vaccine information. Alternatively, some may have decided to postpone taking vaccines from other manufacturers to reevaluate their vaccine choices considering approval of the Pfizer-BioNTech vaccine. The contrasting relative increase of series completions may reflect that approval was associated with the decisions of individuals who forgot or had barriers to scheduling a second appointment or previously had misgivings after taking the first dose.

Our results call for more research into the association of FDA approval with US vaccine uptake. They also call for more work into the distinctions between unvaccinated individuals and those who have received 1 dose to improve our understanding of how interventions could motivate the 2 groups toward full vaccination.

## Notes

Supplement. **eAppendix. **Supplemental Methods
eReference
