# Supplementary material for: Vaccine Uptake in the US After Full Food and Drug Administration Approval of the BNT162b2 mRNA COVID-19 Vaccine
Source: JAMA Netw Open. 2022 Apr 6;5(4):e226108. doi: 10.1001/jamanetworkopen.2022.6108 (PMC8987898; doi:10.1001/jamanetworkopen.2022.6108)
Supplement: Supplement. — eAppendix. Supplemental Methods eReference [file jamanetwopen-e226108-s001.pdf]

Supplemental Online Content

Bailey EV, Wilson FA. Vaccine uptake in the US after full Food and Drug Administration approval of the BNT162b2 mRNA COVID-19 vaccine. *JAMA Netw Open*. 2022;5(4):e226108. doi:10.1001/jamanetworkopen.2022.6108

eAppendix. Supplemental Methods
eReference

This supplemental material has been provided by the authors to give readers additional information about their work.

© 2022 Bailey EV et al. *JAMA Network Open.*

eAppendix. Supplemental Methods

Description of analytic approach

Across all study analyses, we used a statistical method called causal impact analysis, as implemented in R's CausalImpact package. The method is an alternative to interrupted time series modeling that accounts for autocorrelation and seasonality. In this analysis, which was based on bayesian structural time-series modelling, a counterfactual time trend that would have been observed without the impact of an intervention was constructed using a synthetic control. That synthetic control was generated using multiple sources of information: first, pre-intervention values in the outcome time series; second, values from a set of one or more covarying time series; and third, prior knowledge about limited model parameters elicited from the literature or empirically estimated. The covarying series was the time trend of the outcome of interest in a geographical area that did not receive the intervention. This allowed the analysis to account for variance shared by the time series of interest and the covarying series, such as unobserved events shared between the two that would otherwise have gone unaccounted for. As implemented in the package, the handling of the covarying series in the generation of the synthetic composite control was done empirically using spike and slab priors. Once the counterfactual was estimated from the synthetic composite control, the estimated counterfactual outcomes were subtracted from the actual observed post-intervention outcomes to provide an estimate of the estimated change in the outcome that may have resulted from the intervention. 95% credible intervals were generated using Markov Chain Monte Carlo methods.

This analysis included a few important assumptions. First, the covarying series must not have been impacted by the intervention—in this case FDA approval of the vaccine in the U.S. must not have impacted vaccine uptake in the UK. Second, the analysis assumes that the relationship between the covarying series and the series of interest was consistent across both the pre- and post-intervention periods. The third is that the priors were correctly specified. In this case, we used default priors for the package as is common in prior literature using the method. This included standardizing the data before model fitting—an empirical approach to setting priors; the choice of 0.01 for the prior standard deviation of the Gaussian random walk at the local level; and the choice to use static regression coefficients rather than time-varying ones to avoid overspecification of the model.

More details about how the synthetic control was generated and the counterfactual is predicted are available elsewhere.[1]

Use of UK vaccination data

The vaccination trend in the United Kingdom (UK) was used as a covarying series, which was combined with information about the pre-approval trend in the U.S. to create a synthetic control group against which observed U.S. vaccination counts were compared. The UK was selected because it is a large, industrialized country in which the Pfizer-BioNTech vaccine does not account for most vaccines distributed. Furthermore, we believe UK residents may be more likely to respond to approval decisions made by the UK regulatory agency—the Medicines and Healthcare Products Regulatory Agency—than those made by the U.S. FDA. Thus, the likelihood of the UK vaccination trend being affected by announcement of full FDA approval for Pfizer/BioNTech's vaccine is low.

Limitations of this analytic approach

Our analytic approach has limitations. Chief among them is that covarying series in models were assumed to be entirely unaffected by the intervention. This assumption may have been violated to some extent if behavior of individuals living in the UK was impacted by hearing that the FDA had fully approved the Pfizer-BioNTech vaccine for use in the U.S. We do not believe such spillover effects were likely to be present but, if they were, our estimates would be conservative. In addition, the study period was short, particularly the post-intervention period. However, we felt that it was not appropriate to include data after

© 2022 Bailey EV et al. *JAMA Network Open.*

the Biden administration's announcement of potential large vaccine mandates, as the announcement may have impacted vaccine uptake. It is possible that some individuals had insufficient time to act on the FDA approval announcement by September 9th. This issue will make our estimates conservative, but is not expected to differentially impact one group, e.g., those taking their first dose, versus other groups, e.g., those completing their dose series. Furthermore, the US was also experiencing large vaccine surpluses during that period so that vaccine appointment lags were not a significant factor. Finally, it is possible that the rise of the Delta variant of COVID-19 impacted our analyses. However, Delta became the predominant strain in the U.S. as early as mid-July and was widely publicized between then and early August. Because FDA approval occurred in late August, we believe that much of the behavioral response to Delta vis-à-vis vaccinations would have occurred prior to FDA approval.

eReference

1. Brodersen KH, Gallusser F, Koehler J, Remy N, Scott SL. Inferring causal impact using Bayesian structural time-series models. *Ann Appl Stat*. 2015;9(1):247-274. doi:10.1214/14-AOAS788

© 2022 Bailey EV et al. *JAMA Network Open.*
